# Early Parallel Activation of Semantics and Phonology in Picture Naming: Evidence from a Multiple Linear Regression MEG Study

**DOI:** 10.1093/cercor/bhu137

**Published:** 2014-07-08

**Authors:** Michele Miozzo, Friedemann Pulvermüller, Olaf Hauk

**Affiliations:** 1 Johns Hopkins University, Baltimore, MD, USA; 2 Freie Universität Berlin, Berlin, Germany; 3 Medical Research Council, Cognition and Brain Sciences Unit, Cambridge, UK

**Keywords:** language production, lexical access, MEG

## Abstract

The time course of brain activation during word production has become an area of increasingly intense investigation in cognitive neuroscience. The predominant view has been that semantic and phonological processes are activated sequentially, at about 150 and 200–400 ms after picture onset. Although evidence from prior studies has been interpreted as supporting this view, these studies were arguably not ideally suited to detect early brain activation of semantic and phonological processes. We here used a multiple linear regression approach to magnetoencephalography (MEG) analysis of picture naming in order to investigate early effects of variables specifically related to visual, semantic, and phonological processing. This was combined with distributed minimum-norm source estimation and region-of-interest analysis. Brain activation associated with visual image complexity appeared in occipital cortex at about 100 ms after picture presentation onset. At about 150 ms, semantic variables became physiologically manifest in left frontotemporal regions. In the same latency range, we found an effect of phonological variables in the left middle temporal gyrus. Our results demonstrate that multiple linear regression analysis is sensitive to early effects of multiple psycholinguistic variables in picture naming. Crucially, our results suggest that access to phonological information might begin in parallel with semantic processing around 150 ms after picture onset.

## Introduction

In every form of verbal communication, speakers are confronted with choosing the right word at the right time. Much of what we know about the neurocognitive mechanisms of word production has come from picture naming, a task that has represented a model for investigating 3 core processes of word production: 1) semantic access, through which distinguishing semantic features of the depicted concept become available (e.g., the features pet, feline, and purring, for the word “cat”), 2) word form selection, which leads to the retrieval of stored features of word sounds (e.g., the phoneme sequence /kat/), and 3) speech planning and articulation (Levelt et al. 1999). The investigation of event-related potentials (ERPs) and magnetic fields in picture naming has provided an unprecedented opportunity to characterize the time course of activation within the brain network supporting word production (Salmelin et al. 1994; Turennout et al. 1998; Schmitt et al. 2000; Rahman and Sommer 2003; Costa et al. 2009). Questions about the temporal sequence of processes can be answered using electrophysiological methods with millisecond temporal resolution, such as electro- and magneto-encephalography (EEG and MEG). Behavioral responses (e.g., voice onset times or button press latencies) are less suited; as they record the endpoint of a complex chain of processes, they may reflect not only processes of interest (e.g., semantic access) but also processes related to decision-making and response preparation. Metabolic imaging techniques, such as functional magnetic resonance imaging (fMRI), positron emission tomography (PET), or near-infrared spectroscopy, have temporal resolution that is less precise and are therefore equally suboptimal for addressing questions about the precise millisecond time course of picture naming.

There is still controversy in the literature about the precise time course of brain activation during picture naming. The currently most detailed neurocognitive account of word production has been proposed by Indefrey and Levelt (2004) (see also Indefrey 2011). Based on a large body of behavioral studies, as well as a meta-analysis of the existing neuroimaging and electrophysiology literature, they conclude that language production is best described by a serial succession of processing stages: semantic processing starts at about 150 ms (times are from stimulus picture onset), primarily involving activation in left posterior temporal–anterior occipital areas. Word form processing starts slightly later, after an estimated gap of about 50 ms and no earlier than about 200 ms, involving the left posterior superior and middle temporal gyri (but probably also the left anterior insula and the right supplementary motor area). Activation in premotor/precentral cortex and inferior frontal gyrus (IFG) typically appeared at about 400 ms. Such late frontal activation, which could be recorded using MEG, was related to articulatory planning (Levelt et al. 1998; Vihla et al. 2006). However, it has recently been argued that the evidence reviewed by Indefrey and Levelt (2004) may not provide a precise time frame of picture naming (Strijkers et al. 2011). These authors argue that some of the critical electrophysiological studies did not use experimental manipulations that allow accurate conclusions about the underlying processes and that results could reflect response strategies rather than the time characteristics of naming responses. This issue is discussed in more detail later.

Investigating the early components of cortical evoked responses is challenging, because early neurophysiological brain responses are known to be generally focal and short-lived, in contrast to late ones that are typically more global, long-lasting, and sometimes large in size, especially in the language domain (for review, see Pulvermuller et al. 2009). Therefore, the more localized and the earlier these effects are, the smaller we should expect their effect size to be. Sensitive statistical methods that optimally exploit the information available in the data are needed to investigate the early brain mechanisms involved in word retrieval. As we will describe in detail later, this objective was pursued here applying multiple linear regression in combination with distributed source estimation to MEG responses evoked by spoken picture naming. This approach was previously used in ERP research on word recognition (Hauk et al. 2006, 2009), and it is being extended to MEG for the first time in this present work.

### Relative Timing of Semantic and Phonological Processes

The predominant view held by current accounts of word production is what we refer to as the “Semantic Priority hypothesis” (Dell 1986; Butterworth 1989; Glaser 1992; Caramazza and Miozzo 1997; Levelt et al. 1999; Rapp and Goldrick 2000; Sahin et al. 2009). According to this hypothesis, access to semantic information precedes access to information specifying other features of words in the object-naming process. The nature of this information has remained an issue of debate. Caramazza and Miozzo (1997) and Harley and Brown (1998) proposed that semantics directly activates word forms, whereas other proposals assume an intermediate lexical representation (often referred to as “lemma”; Levelt et al. 1999) in between semantics and word form representations that encodes the syntactic features of words (e.g., grammatical class). Because all proposals agree that access to semantic information precedes access to word forms, we examined and tested this more inclusive formulation of the Semantic Priority hypothesis. It should be emphasized that the hypothesis concerns the sequence with which semantic and word form information is accessed, and not the time it takes to select such information. Here, we also examine an alternative proposal, the “Simultaneous Ignition hypothesis.” Under this proposal, visual object recognition triggers the simultaneous retrieval of both semantics and form when a speaker is engaged in naming.

So far, most MEG and EEG studies have been interpreted as supporting the Semantic Priority hypothesis. In their pioneering MEG study, Salmelin et al. (1994) reported activation spreading from posterior to frontal brain areas within about 400 ms. This was interpreted as reflecting semantic followed by phonological processing. However, such an interpretation was based on the localization of MEG signals averaged across the whole stimulus set, without using experimental manipulations linking MEG sources to semantic or phonological processes. A similar approach was undertaken in a subsequent study conducted by Levelt et al. (1998). Similar limitations apply to results from intracranial recordings of brain activity (Llorens et al. 2011) typically conducted on patients suffering from drug-resistant epilepsy, which revealed activation spreading with the same general spatio-temporal characteristics demonstrated in most of the MEG and EEG studies.

In a different line of research, language tasks were varied to investigate the time course of psycholinguistic variables affecting word production. Several studies looked at the relative timing of semantic, syntactic, and phonological processes as indexed by the lateralized readiness potentials (LRPs) (Turennout et al. 1997, 1998). These studies used dual-task paradigms, where one type of information (e.g., semantic) determines whether a response is required or not, and another type of information (e.g., phonological) whether the response should be given with the left or right hand. Differences in onset latencies of the LRP depending on task requirements were interpreted as reflecting differences in the timing of semantic, syntactic, and phonological processes (for converging EEG results, see Jescheniak et al. 2002; Rodriguez-Fornells et al. 2002; Schmitt et al. 2000). However, it has been argued that these results may reflect late response strategies rather than early object processing (Strijkers et al. 2011), and it has been shown that the pattern of results strongly depends on the choice of the information determining the response (Rahman and Sommer 2003). Any conclusions on the precise timing of the brain indexes of access processing in picture naming must therefore remain tentative until methods are available to address the issue in a more direct manner.

Such a method may be provided by multiple linear regression applied to neurophysiological signals (Hauk et al. 2006). We here applied this new method to study the spatio-temporal dynamics of language production measuring brain responses during overt picture naming and analyzing several specific psycholinguistic variables that reflect the different processes involved in picture naming (visual, semantic, and phonological).

Psycholinguistic research has identified a variety of variables putatively affecting specific processes supporting picture naming. For example, number of semantic features has typically been related to semantic processing, whereas number of phonemes contained in picture names to word form processing (Snodgrass and Vanderwart 1980; Bates et al. 2003; Alario et al. 2004; McRae et al. 2005). Multiple linear regression is well-suited for analyzing the effects of multiple variables for several reasons (e.g., Baayen 2004):

1. It allows using continuous information in the predictor variables and does not require an artificial binary categorization of the stimuli (e.g., into high- and low-frequency words). This can lead to higher statistical sensitivity.
2. Because it does not require categorization of the stimulus set, it avoids the potential problem of choosing “awkward” stimulus items at extreme ends of the parameter distribution during stimulus matching (e.g., very high- or very low-frequency stimuli).
3. Multiple linear regression can deal with moderate degrees of collinearity among the predictor variables. It is therefore a flexible method with respect to the inclusion of experimental items.

### Testing the Semantic Priority and Simultaneous Ignition Hypotheses

The temporal relationship between semantic and word form processing in picture naming can be straightforwardly investigated using multiple linear regression by comparing the point in time at which the effects of predictor variables associated with each of these processes appear. We here used this approach to explore the neurophysiological manifestations of compound variables reflecting visual, semantic, and lexical/phonological features associated with the pictures presented for naming and the words typically used to name the depicted objects. These variables are presented in detail in the Methods section. Based on previous research and our specific hypotheses, the following predictions were tested: according to behavioral and ERP research on object categorization (e.g., Thorpe et al. 1996, 2001), as well as the meta-analysis by Indefrey and Levelt (2004) and Indefrey (2011), we expected semantic features to affect the brain responses around 150 ms. Critically, according to the Semantic Priority hypothesis, effects of word form features should be distinct from and follow those of semantic features. In contrast, the Simultaneous Ignition Hypothesis predicts immediate near-simultaneous effects for these variables at an early latency, within the first 200 ms upon object presentation.

In our MEG experiment, we used overt naming of familiar objects, a task speakers naturally use in everyday life and therefore is unlikely to engender artificial (task-specific) response strategies. However, the use of this task restricted our analysis to the latency range prior to the earliest articulatory movements that would alter MEG recording, (approximately up to about 300 ms). We therefore focused on the earliest effects of the predictor variables we tested. We use distributed minimum-norm estimation (MNE) to determine the main neuronal sources of the effects of the predictor variables. In addition, we employed a theory-guided region-of-interest analysis, which allowed an analysis of the brain dynamics during picture naming in space and time.

## Methods

### Participants

Participants (*N* = 17, 11 females, age range: 20–30 years) self-reported British English as their native language and the only language they speak fluently and regularly. All had normal or corrected-to-normal vision, reported no history of neurological illness or drug abuse, and were right-handed (as determined by a 10-item version of the Oldfield (1971) handedness inventory). Participants were paid for their participation. Informed consent was obtained from all participants. The study received approval from the Cambridge Psychology Research Committee.

### Picture Stimuli

We selected 146 line drawings from the Snodgrass and Vanderwart (1980) set. The drawings depicted familiar objects from a broad range of semantic categories and obtained high name agreement rates in British English (mean = 94%, range = 77–100%; Barry et al. 1997). The names most commonly assigned to the pictures (Barry et al. 1997) were monomorphemic, with the exception of the transparent compounds “motorbike, ashtray, record player and waistcoat,” and the derived noun “toaster.”

### Predictor Variables

Four variables were used as predictors of the multiple correlation analyses conducted on MEG data and were labeled as Visual Complexity, Specific Semantic Features, Action Features, and Word Form, respectively. A predictor value was obtained for each variable for each picture named in the study. In the first part of this section, we describe the rationale that guided the selection of 3 of these predictor variables (Visual Complexity, Specific Semantic Features, and Action Features); next, we describe how the individual predictor variables were obtained.

#### Semantic Predictor Variables

Identifying the semantic predictor variable is not trivial because semantics is a multi-faceted system, with semantic features differing for modality and content possibly being related to different brain regions and access times (Barsalou et al. 2003; Damasio et al. 2004; Pulvermuller et al. 2005; Martin 2007; Patterson et al. 2007; Binder et al. 2009; Bedney and Caramazza 2011). An issue of debate has concerned whether sensory and motor areas contribute to semantic processing providing information that is integral to meaning. In recognition of this debate, we used 2 distinct semantic variables. One predictor variable, Specific Semantic Features, refers to the distinguishing features of a concept (e.g., “moo” for “cow”) that are supposedly critical for the identification of a specific concept (Tversky 1977; Cree and McRae 2003; Cree et al. 2006). The other predictor variable, Action Features, relates to actions afforded by an object and likewise viewed under some proposals (e.g., Barsalou et al. 2003; Pulvermuller et al. 2005) as an integral component of word meaning. Importantly, Specific Semantic Features and Action Features refer to properties of concepts not explicitly depicted in the pictures showed to participants. This is not the case with semantic information corresponding to visual features, and this was the reason for not using a semantic predictor related to visual features. We also excluded predictor variables associated with other sensory features (e.g., auditory or tactile) because many objects might not be characteristically associated with features of this kind (Cree and McRae 2003). It should be emphasized that the inclusion of the predictor variable Action Features is orthogonal with respect to the debate about the nature of semantics. Our data help us determine the extent and time course of brain activation induced by the action features of the depicted objects, not whether action features form an integral component of semantics. Nevertheless, including the predictor variable Action Features, we achieve a more comprehensive characterization of the neural network involved in picture naming.

#### Visual Complexity

Although our primary interest was on semantic and phonological processes, it is important to include a visual variable in order to characterize the time course of the visual processing implicated in picture naming. The visual variable serves as a proof of concept, in that we can strongly expect early reliable effects of this variable in posterior visual brain areas around 100 ms. These results can also serve as a time marker, since we can assume that the earliest effects of semantic and lexical/phonological variables follow those of the visual variable. The visual variable we used—Visual Complexity—refers to measures of the complexity of the named pictures, as explained in detail later.

#### Principal Component Analysis

Several measures of specific semantic, action-related, and word form features are available for the pictures from Snodgrass and Vanderwart (1980) that were named in our study. In principle, it would be possible to enter all of these measures into 1 regression model simultaneously. However, this approach results in larger data processing demands as well as less sensitive analyses, especially if highly correlated variables “compete” to explain the same unique variance. Therefore, we efficiently reduced the number of measures using PCA (for a similar procedure, see Hauk et al. 2009) (PCA was not carried out for Visual Complexity since this variable corresponded to a single measure). The selection of the predictor variables Specific Semantic Features, Action Features, and Word Form proceeded in 2 steps. In a first step, for each picture, we identified published measures corresponding to specific semantic, action-related, and word form features, respectively. Measures corresponding to each of these features were entered in distinct PCAs that yielded 3 distinct predictor variables for each picture: Specific Semantic Features, Action Features, and Word Form. As in Hauk et al. (2009), the first principal component obtained for each picture in each of these analyses was used as predictor variable. The first principal component values entered in the multiple correlation analyses were standardized—hence, they had mean equal to 0 and SD to 1 and ranged between about −2 and 2.

#### Variable Components

Below, we describe the specific measures used to derive each of the 4 predictor variables.

1. Visual Complexity corresponds to ratings of the complexity of a drawing (Snodgrass and Vanderwart 1980), as defined by the number of lines in a drawing and their intricacy.
2. Specific Semantic Features was composed of 2 measures grouped through PCA. One measure refers to distinguishing features, operationalized as the number of features a concept shares with only few other concepts (e.g., “moos/cow”). The other measure pertains to the number of encyclopedic features, defined as those features relating neither to sensory nor functional aspects of concepts (e.g., “knife/dangerous”). Both of these measures were obtained by McRae et al. (2005) by tallying the different types of features included in written descriptions of concepts provided by a large group of English speakers. These 2 measures are weakly correlated with each other (*r* = 0.14, *t*_(144)_ = 1.79, *P* = 0.07).
3. Action Features was obtained through PCA grouping 2 measures of actions associated with an object. One measure was also collected by McRae et al. (2005) and refers to ways in which people interact with objects (“knife/cutting”). The other measure (Magnie et al. 2003) is a rating of the ease with which related actions are mimed in response to the same object pictures presented in our study. These 2 measures are strongly correlated with each other (*r* = 0.49, *t*_(144)_ = 6.87, *P* < 0.0001).
4. Word Form was obtained from 2 measures of picture names in British English: word length (number of phonemes) and number of phonological neighbors (words of the same length as a picture name but differing by 1 phoneme; norms from Balota et al. 2007). Effects of neighborhood size have been demonstrated in word production and explained as reflecting activation spreading to words phonologically related to the target—for example, the activated neighbors of “cat” include “cot, bat, mat, etc.” (Harley and Brown 1998; Vitevitch 2002; Dell and Gordon, 2003). Word length and neighborhood size are strongly correlated with each other (*r* = −0.68, *t*_(144)_ = 11.24, *P* < 0.0001). Because word length and neighborhood size are strongly correlated with word frequency (rs = −0.51 and 0.46, respectively), log-transformed frequencies (from CELEX, Baayen et al. 1993) were also included in the PCA that yielded the Word Form predictor.

Examples of pictures with high and low coefficient values for each of the predictor variables are shown in Table 1.

**Table 1:** Examples of pictures with high/low coefficient values for each predictor variable

Predictor variable	Coefficient values	
	High	Low
Visual Complexity	Motorbike, peacock, piano	Pear, box, skirt, envelope giraffe
Specific Semantic Features	Tiger, potato, cake, lion	Pipe, bow, harp, ball
Action Features	Screwdriver, shoe, gun, spoon	Swan, ant, leopard, pineapple
Word Form	Door, car, chair, tie	Caterpillar, screwdriver, helicopter, strawberry

Correlation coefficients between predictor variables and measures from which predictor variables were derived are shown in Table 2. Predictor variables were correlated strongly with the measures from which they were derived (mean *r* = 0.87) but weakly with the other measures (mean = 0.17; *t*_(21)_ = 15.47, *P* < 0.0001). Although multiple linear regression can deal with collinearity of predictor variables, it is desirable that predictor variables are weakly correlated with each other. This held among our predictor variables (*r* = 0.09–0.23; see Table 2), with the exception of the visual and action predictors that were moderately correlated (*r* = −39).

**Table 2:** Correlation coefficients between predictor variables and variable components

Predictor Variable	Predictor variable							
	Visual Complexity	Specific Semantic Features	Action Features					
Visual Complexity	—							
Specific Semantic Features	0.12	—						
Action Features	−0.39	−0.20	—					
Word Form	−0.23	08	0.19					
Predictor Variable	Variable Components/Type							
	Visually related	Semantically related			Action related	Word Form related		
	Complexity	Distinct Features	Encyclopedic Features	Number Actions	Mimed Actions	Word Frequency	Number Phonemes	Number Neighbors
Visual Complexity	**1.0**	0.09	0.11	0.32	−0.35	−0.24	0.14	−0.21
Specific Semantic Features	0.12	**.084**	**0.84**	0.06	−0.29	0.19	−0.02	0.02
Action Features	−0.39	−0.01	−0.33	**0.86**	**0.86**	0.22	−0.09	0.17
Word Form	−0.23	0.16	−0.01	0.12	0.20	−**0.77**	**0.89**	**0.88**

Correlation coefficients with the predictor variables in bold.

### Picture-Naming Task

All stimulus pictures were shown as black line drawings on a white background and were projected on a screen covering an area within about 4° × 4° of the central visual field. In each trial, the fixation point (a cross) was shown for 600 ms and immediately replaced by a picture, which remained in view for 600 ms. Inter-trial intervals varied between 2.4 and 2.8 s. Order of presentation was randomized and differed across participants. E-prime (Psychological Software Tools, 2002) was used for picture presentation and naming latency recording. Naming latencies were determined as the time elapsing between picture onset and the beginning of vocalization and were recorded on line using OPTIMIC™ optical microphone. Participants were instructed to verbally name the pictures as fast as possible without hesitations or stammering. Participants named the whole picture set before the experiment to familiarize with the materials and a second time during the experiment proper. No feedback was provided to participants about the names they selected for the pictures.

### MEG: Recording Procedure

MEG was measured in the magnetically shielded MEG booth at the MRC Cognition and Brain Sciences Unit in Cambridge, UK, using the 306-channel Neuromag Vectorview system (Elekta AB), and combining 204 planar gradiometers and 102 magnetometers at a sampling rate of 1000 Hz. The following positions were digitized before the MEG recording session for movement compensation in the Maxfilter software (below) and for accurate coregistration with MRI data using a 3Space Isotrak II System: 1) 5 head position indicator coils, 2) 3 anatomical landmark points (nasion and preauricular points), and 3) 50–100 additional randomly distributed points (head shape). The electro-oculogram (EOG) was recorded bipolarly through electrodes placed above and below the left eye (vertical EOG) and at the outer canthi (horizontal EOG).

### MEG Data Analysis: Preprocessing

In a first step, the signal-space separation (SSS) method implemented in the Neuromag Maxfilter software (version 2.0) was applied to our MEG data in order to remove artifacts likely to arise from sources distant from the sensor array. The spatio-temporal variant of SSS as well as movement compensation was applied, and statically bad channels were interpolated. Data were then band-pass-filtered between 0.1 and 40 Hz using the MNE software (http://www.nmr.mgh.harvard.edu/martinos/userInfo/data/sofMNE.php) and the procedure described in Gramfort et al. (2014). For averaging and regression analysis, data were divided into epochs of 600 ms length, starting 100 ms before picture onset and ending at 500 ms after picture onset. Epochs terminated before the onset of most of the spoken responses to avoid artifacts generated by articulatory movements. Epochs were rejected when maximum–minimum amplitudes in the 100- to 500-ms interval exceeded the following thresholds: 150 µV in the EOG, 2500 fT in magnetometers, and 1000 fT/cm for gradiometers. After removing trials with errors and artifacts, an average of 121 trials (SD = 23) per participant were entered in analyses.

### Regression Analysis of MEG Data

Regression analysis was applied in the same way as described previously for EEG data (Hauk et al. 2009). Solving the linear-regression equation **y**= **Xb**, where **y** represents the vector of measurements at 1 sensor and for 1 post-stimulus latency across *n* trials, **X** is the *n*-by-4 design matrix containing the 4 predictor variables (Visual Complexity, Specific Semantic Features, Action Features, and Word Form) and **b** includes the 4 event-related regression coefficients (ERRCs) that reflect the relationship between predictor variables and data (i.e., 1 element for each predictor variable). The solution of this equation results in a linear estimator **G** (4-by-*n*) for each predictor variable, which is multiplied with the data **y** in order to obtain the ERRCs **b** for a particular channel and latency. This means that for each channel, latency, and predictor variable, the resulting ERRC is a weighted average of all trials, where the weighting coefficients are determined by the linear estimators in **G**. Baseline correction with respect to a 100-ms pre-stimulus interval was applied to each epoch before applying the weightings. An important feature of ERRCs is that source analysis can be applied to these data as for conventional averages or difference data (Hauk et al. 2006). Furthermore, this procedure allows using the same response set across predictor variables.

It is important to note that linear regression analysis is an extension of—but not fundamentally different from—traditional factorial analysis. A factorial analysis can be described as a special case of regression, in which, for example, all items of 1 category are weighted by a factor 1/*n*, and the other category by −1/*m* (where *n* and *m* are the numbers of items within the categories, respectively). In our regression analysis, these values vary from item to item depending on the predictor variables. The resulting ERRCs can therefore be interpreted and analyzed similarly to the results from traditional subtraction designs.

The main conclusions of our study will be drawn from region-of-interest analyses in source space. However, in ERP studies, it is standard to present data also in signal space (Picton et al. 2000).

In order to describe the time course of our data and to determine peaks and latency ranges of interest, we displayed the root-mean-square (RMS) of the signal-to-noise ratio (SNR) across all magnetometers and gradiometers. The computation of SNRs prior to RMS is useful because it renders the values for all channels unitless (original measurements are in T and T/m, respectively) and allows the computation of a combined measure for display. This is appropriate since source analysis was computed from the combination of all sensors as well.

### Source Estimation

**Figure 1.**
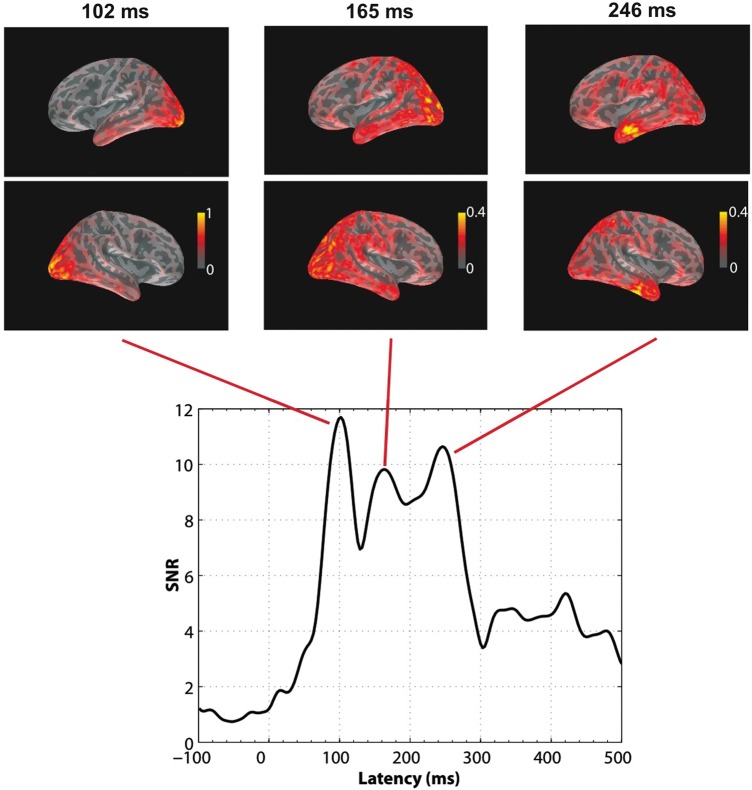
Time course of SNR computed as the RMS of SNRs across all sensors, on the grand mean obtained from all pictures during the recording epoch (0 ms = picture onset). Three peaks were observed, at 102, 165, and 246 ms, respectively. Grand average dSPM snapshot of activation obtained with MNEs at those peak latencies are presented for the left and right cerebral hemispheres on inflated cortical surfaces showing sulci as darker gray areas and gyri as lighter areas. Color scales indicate dSPM values. Note the different scales. Activation shows a posterior–anterior direction of spreading within the 100- to 300-ms interval.

**Figure 2.**
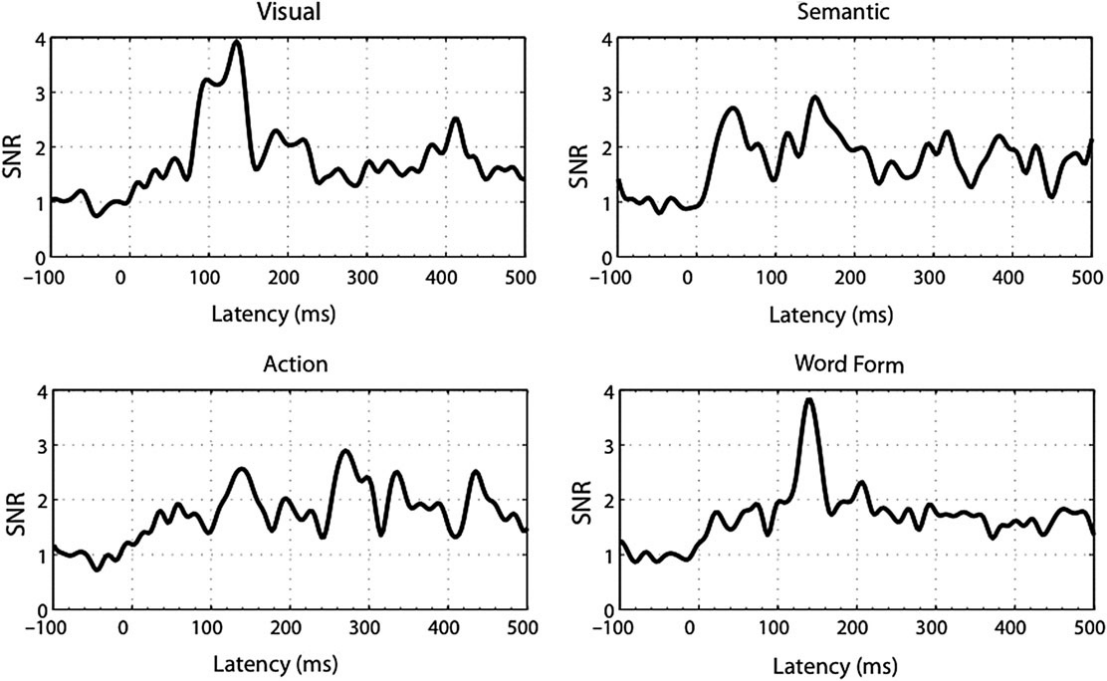
Root-mean-square of SNRs (similar to Fig. 1) for ERRCs of individual predictors (Visual Complexity, Specific Semantic Features, Action Features, and Word Form; 0 ms = picture onset). The visual predictor showed a first peak at 96 ms. The semantic, action, and word form predictors had peaks at about 150 ms.

Distributed minimum-norm source estimation was applied following standard procedure in the MNE software package (http://www.nmr.mgh.harvard.edu/martinos/userInfo/data/sofMNE.php). Minimum-norm estimation makes minimal assumptions about the structure of the brain generators (e.g., their number or locations) and is optimal for the analysis of complex or completely unknown source configurations within the general resolution limits of MEG measurements (Hämäläinen and Ilmoniemi 1994; Hauk and Pulvermuller 2004, Hauk et al. 2011). The noise covariance matrices were computed for each experimental session since noise levels may change between sessions. However, source estimates for different predictors were based on the same noise covariance matrices that were computed for baseline intervals of 200 ms duration before picture onset. For regularization, we specified an SNR of 3 in the MNE software. The choice of SNR = 3 approximately corresponds to 10% of unexplained variance, which, based on the results in Figure 1, is a realistic estimate for the peak latencies presented in Figure 2.

MEG sensor configurations and MRI images obtained for each participant were co-registered based on the matching of 1) digitized locations on the scalp surface (varying in number between ∼50 and 100 across participants) with 2) the reconstructed scalp surface from the FreeSurfer software (see below). High-resolution structural T1-weighted MRI images were acquired in a 3T Siemens Tim Trio scanner at the MRC Cognition and Brain Sciences Unit in Cambridge (UK) using a 3D MPRAGE sequence, field-of-view 256 × 240 × 160 mm, matrix dimensions 256 × 240 × 160, 1 mm isotropic resolution, TR = 2250 ms, TI = 900 ms, TE = 2.99 ms, flip angle 9°. Structural MRI images were processed using automated segmentation algorithms of the FreeSurfer software (Version 4.3; Fischl et al. 2001). The result of the FreeSurfer segmentation was processed further using the MNE software package (Version 2.6). The original triangulated cortical surface (consisting of several hundred thousand vertices) was down-sampled to a grid using the traditional method for cortical surface decimation with an average distance between vertices of 5 mm, which resulted in approximately 10 000 vertices. A boundary element model containing 5120 triangles was created from the inner skull surface, which was generated using a watershed algorithm. Dipole sources were assumed to be perpendicular to the cortical surface. Source estimates were computed for each participant and predictor variable. The individual results were morphed to the average brain across all participants, and grand-averages were computed. These grand-averages were then displayed on the inflated average cortical surface.

### ROI-Driven Analyses

**Figure 3.**
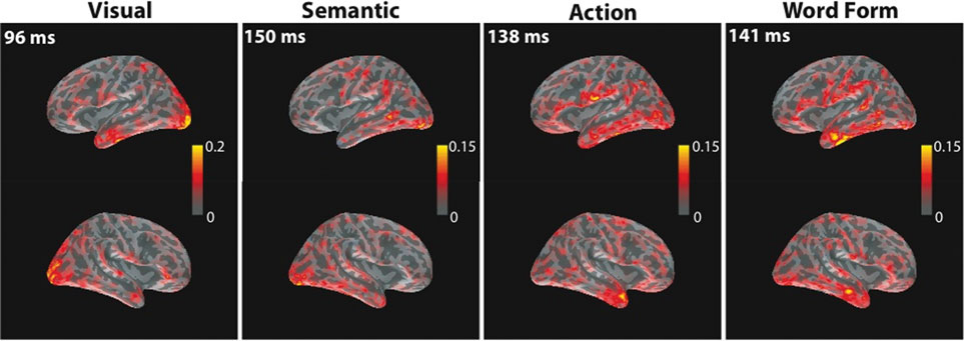
The MNEs at peak latencies corresponding to each predictor (Visual Complexity, Specific Semantic Features, Action Features, and Word Form) as revealed by the ERRCs reported in Figure 2. MNEs are shown for the left and right cerebral hemispheres on inflated cortical surfaces. Color scales indicate ERRC values. Note the different scales.

ROIs analyses were conducted on 6 left hemisphere cortical areas that prior studies have associated with the specific processes investigated in the present study. The occipital ROI was chosen because of its involvement in visual processing. The middle and inferior temporal cortex has consistently been shown to be involved in semantic processing (Indefrey and Levelt 2004; Vigneau et al. 2006; Mechelli et al. 2007; Binder et al. 2009), and effects of Specific Semantic Features were expected especially in ROIs extending over these temporal regions. With respect to Action Features, we focused on the ventral premotor cortex (vPMC), one component of the brain network processing knowledge about actions (Boronat et al. 2005; Saccuman et al. 2006; Beuchanp and Martin 2007; Rueschemeyer et al. 2010), which in turn is part of a larger temporal-frontal network supporting object knowledge (Martin 2007; Rizzolatti and Sinigaglia 2010). Left cortical areas previously associated with word form processing include middle temporal and posterior superior temporal (Indefrey and Levelt 2004; Graves et al. 2007; Wilson et al. 2009; Peramunage et al. 2010), and effects of Word Form were anticipate in ROIs centered in these areas. An additional ROI was localized in inferior frontal cortex, a region that has been suggested to play a role in phonological, semantic, and action-related processing (e.g., Bookheimer 2002; Pulvermuller and Fadiga 2010; Rizzolatti and Sinigaglia 2010). This ROI was chosen not only to ascertain whether effects of semantic, action-related, and/or word form predictors appear in IFG but also to determine whether activation in picture naming spreads from posterior to frontal brain regions within approximately 100- to 200-ms time interval. ROIs were defined based on the source estimates for the average across all pictures (i.e., on a condition orthogonal to the predictor variables) (Fig. 3).

**Figure 4.**
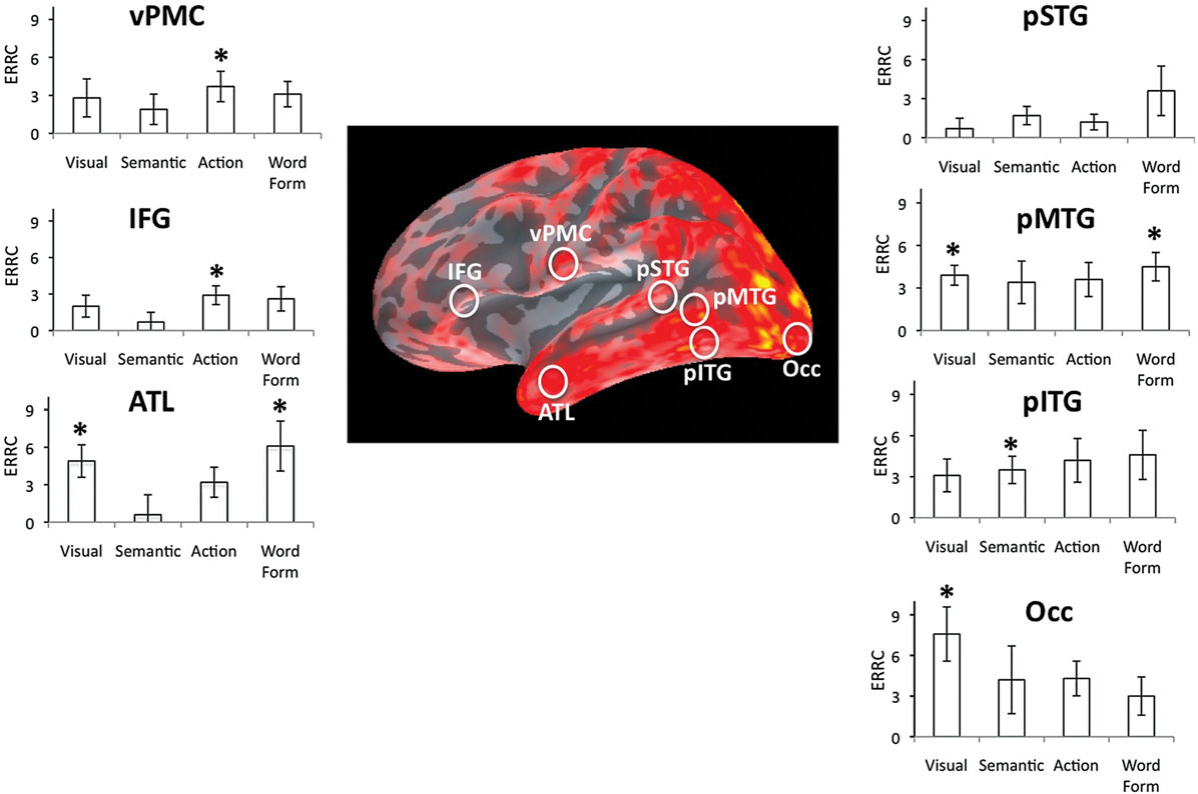
Regions of interest tested with the 4 predictors (Visual Complexity, Specific Semantic Features, Action Features, and Word Form) during the 130- to 160-ms interval. ROIs were localized in the following left hemisphere areas: occipital (Occ), posterior inferior temporal gyrus (pITG), posterior middle temporal gyrus (pMTG), posterior superior temporal gyrus (pSTG), inferior frontal gyrus (IFG), and ventral premotor cortex (vPMC). ROIs are projected on the grand average dSPM snapshot of activation in left hemisphere at 160 ms (the color scale indicates dSPM values). For each predictor and each ROI, graphs illustrate the ERRCs averaged across the 130- to 160-ms time interval. Vertical bars correspond to standard errors. Asterisks indicate ERRCs with values significantly greater than 0 at a specific location (*P* = 0.007 corrected for testing of multiple loci).

Our main analysis focused on regions of interest described in previous literature, such as the meta-analysis of Indefrey and Levelt (2004). Most of previous localization results are based on fMRI or PET data. These should not be directly imposed on source estimates from EEG/MEG data, since the latter have limited spatial resolution and are prone to systematic mis-localization (Molins et al. 2008; Hauk et al. 2011). We therefore defined the general regions of interest based on previous literature (e.g., middle temporal lobe) but selected the precise ROI for our data analysis based on the most prominent activation peak within this region in our brain-wide source estimates obtained for all participants and all pictures averaged together (ROIs are illustrated as white circles in Figure 4). Specifically, ROIs were localized on cortical regions where, upon visual inspection using the mne_analyze function in the MNE software package, we observed the greatest activation throughout the recording epoch. Note that comparisons of amplitudes between ROIs are usually not informative because they may simply reflect different sensitivities of the sensor configuration to different brain areas. Our hypotheses were therefore tested for ROIs separately. Specifically, intensity values for each ROI and each ERRC were tested within time intervals of theoretical significance and subjected to two-tailed *t*-tests against the zero distribution. A significant result indicates that the predictor variable significantly modulates the brain activity in a specific ROI. Because intensity values are biased toward a non-zero (positive) mean, the average value in the latency range −50 to +50 ms was subtracted before analysis. A baseline interval extending beyond stimulus onset was chosen in order to be temporally closer to the effects of interest and to allow for some variation in noise levels over time—the analysis is therefore more conservative than a pre-stimulus baseline.

## Results

### Behavioral Results

Responses that were excluded from naming latency analyses as well as MEG analyses included: 1) other names than those listed in Barry et al. (1997) (3.8%), 2) responses preceded by hesitations or stuttering (0.8%), 3) exceedingly fast (>300 ms) or slow (<2 s) response onsets (2.9%), and 4) outliers (responses exceeding a participant's mean latency by >2.5 SD; 2.1%). After these exclusions, the number of remaining responses was equal to 2244. Mean naming latency was equal to 722 ms (SD = 197). Entered in a multiple regression, the 4 variables (Visual Complexity, Specific Semantic Features, Action Features, and Word Form) proved to be significant predictors of correct naming latencies (*F*(4, 141) = 5.58, *P* = 0.0003). Furthermore, as revealed by a stepwise regression, Word Form was the only variable that reached significance (*P* = 0.05; *t* = 4.30, *P* < 0.0001). This result possibly reflects the fact that Word Form is the variable most closely associated with naming latencies, a measure of the time it takes to start producing word forms.

### Picture-Evoked Magnetic Brain Responses

To provide an overview of the brain activity generated by picture naming, we analyzed the event-related fields averaged across all pictures and participants (grand mean). As evident from Figure 1, which shows the SNRs derived for the grand mean (gradiometers and magnetometers combined), pictures generated a first prominent peak at about 100 ms. SNRs revealed 2 additional prominent peaks with maxima at 165 and 246 ms, respectively. Overall, picture naming seems to generate the largest activity in the 100- to 300-ms time-window. The distributed MNE maps presented in Figure 1 show the picture-evoked brain responses unfolding in the 100- to 300-ms interval. At 102 ms, activation was mostly confined to bilateral posterior occipital areas. At 165 ms, activation had already spread anteriorly in both hemispheres, particularly over the anterior occipital area and the inferior and middle region of the posterior temporal area, and further activation also appeared in parietal cortex and inferior and superior frontal cortex. At 246 ms, activation in anterior temporal, inferior frontal, and middle prefrontal areas increased.

### Predictor Effects Revealed by Event-Related Regression Coefficients

We obtained ERRCs for each of the 4 predictors (Visual Complexity, Specific Semantic Features, Action Features, and Word Form). The significance of ERRCs was tested against zero in both signal and source space. The time courses of activations for individual ERRCs are displayed in Figure 2 as SNRs across all sensors, similar to Figure 1. The ERRC of the visual predictor exhibited the earliest peak, at 96 ms, as expected. The ERRCs of the semantic, action, and word form predictors showed peaks around 150 ms. These results suggest a processing of semantic features, action knowledge, and word forms starting already around 150 ms.

### ROI Analyses of Cortical Source Dynamics

To localize and further assess cortical activation associated with each predictor, ERRCs were submitted to source estimation, and the resulting local activations were analyzed within pre-specified ROIs (shown in Fig. 4). Figure 2 shows that the largest SNR values were present around 150 ms. Because a latency interval around the largest SNR values is most likely to yield reliable source estimates, localizations were obtained and analyzed within a 130- to 160-ms window.

The predictor effects on ERRC sources were tested at each ROI against the zero distribution using *t*-tests with *P* = 0.008 (corrected for the 6 loci examined with each predictor). Significant effects appeared at the following locations for specific predictor variables:

– in the occipital area for Visual Complexity (*t*_(16)_ = 3.67, *P* = 0.002);
– in the posterior inferior temporal area for Specific Semantic Features (*t*_(16)_ = 3.53, *P* = 0.004);
in the vPMC (*t*_(16)_ = 3.12, *P* = 0.006) and IFG (*t*_(16)_ = 3.77, *P* = 0.001) for Action Features;
in the posterior middle temporal area for Word Form (*t*_(16)_ = 3.92, *P* = 0.001) and Visual Complexity (*t*_(16)_ = 5.59, *P* < 0.001).

No other significant effects occurred in this early latency range. The entire data set is presented in Figure 4. Overall, the significant effects observed in the 130- to 160-ms interval demonstrate considerable time overlap in the processes supporting the access to information about meaning, actions related to objects, and word forms.

## General Discussion

We investigated the spatio-temporal dynamics of language production in an overt picture-naming task employing multiple linear regression analysis of MEG data. Our regression model contained 1 variable describing visual complexity of the pictures, 2 variables describing specific and action-related semantic properties, respectively, and 1 variable accounting for aspects of the form of picture names. Visual Complexity produced noticeable effects already around 100 ms in occipital brain areas, as expected. Crucially, at the early latency of 150 ms, left-hemispheric perisylvian brain regions revealed simultaneous effects of semantic and phonological variables. This matches the predictions of the Simultaneous Ignition hypothesis but contradicts those of the Semantic Priority hypothesis. A further noteworthy finding was that effects were observed in brain loci that previous research linked to the processes corresponding to the individual predictors (the posterior inferior temporal area for the semantic predictor, the vPMC and IFG for the action predictor, and the posterior middle temporal area for the word form predictor). This consistency of localization patterns provides cross-validation for our MEG multiple correlation data.

Altogether, our results suggest several major conclusions. First, there were indications that semantic and phonological features were retrieved closer in time than previously assumed. Second, we observed a fast activation of a left frontotemporal network supporting the processing of meaning and word forms in language production. A third conclusion concerns the localization of 2 different kinds of semantic effects: within 200 ms from picture presentation, activation started to appear not only in posterior inferior temporal areas responding to distinguishing semantic features but also in the IFG and the vPMC where activation was correlated with the degree to which objects are related to action knowledge (Grafton et al. 1997; Chao and Martin 2000; Pulvermuller et al. 2005; Saccuman et al. 2006). The final conclusion is methodological in nature: our results further confirm that multiple linear regression is a sensitive tool for the analysis of specific early brain mechanisms in language processing. This had been shown in previous studies on word perception and comprehension (Hauk et al. 2006, 2009), and here we extend this conclusion to language production in picture naming. The implications of these major results are discussed in the remaining sections of the General Discussion.

### Simultaneity of Semantic and Lexico-Phonological Access in Picture Naming

We found early effects (∼150 ms) of the Word Form variable in posterior middle temporal cortex, the brain area where the effect was expected based on previous neuroimaging studies of verbal object naming. There are converging findings suggesting early activation of word forms near the cortical regions where effects of the Word Form predictor were observed. In picture naming, Strijkers et al. (2010) found that ERPs started to diverge between pictures with high- versus low-frequency names within 200 ms after picture presentation. The time of this word frequency effect is close to the time point where we found that of our Word Form predictor, a composite variable that also included word frequency. Furthermore, effects of frequency and phonological neighborhood—2 of the variables included in the Word Form predictor—had their loci in posterior temporal areas in fMRI investigations of picture naming (Graves et al. 2007; Wilson et al. 2009; Peramunage et al. 2010), thus in close proximity to the middle temporal area where effects of the Word Form predictor appeared in MEG. There are similarities also between the time courses of the effects of the Word Form predictor and the ERP correlates of cognates recorded in picture naming with Spanish–Catalan bilinguals (Strijkers et al. 2010). Whether the name of a picture is a cognate and thus sounds similarly between languages—as “libro/libre,” the translation of “book” in Spanish and Catalan—is a feature of the word related to phonological form. The finding that ERPs started to diverge within 200 ms after picture presentation could be interpreted as suggesting an early activation of word forms.

The precise points in time at which activation associated with visual, semantic, and word form processing appeared in our study might in part reflect specific characteristics of the naming task we adopted. These time markers might change slightly as naming conditions vary. In particular, intention to speak and repetition are both features that likely affected these time markers. ERP results from Strijkers et al. (2011) highlight the relevance of intention to speak. Strijkers et al. (2011) found event-related brain potentials indexing word frequency appearing at about 150 ms in picture naming and 200 ms later in picture categorization, a task in which intention to speak is lacking. To the extent that frequency effects are linked to lexical processing (semantic and/or phonological), the earlier onsets of frequency effects in naming were interpreted as reflecting top-down influences that result in the pre-activation of the neural network implicated in naming and ultimately faster neuronal responses in tasks with explicit intention to speak (Strijikers et al. 2011). Similar forms of top-down pre-activation likely contributed to our MEG findings of early semantic and phonological effects in picture naming. Naming repetition is a further aspect of our study that deserves attention. It should be recalled that pictures were named twice—before the experiment to familiarize participants with the material and during the experiment. Repetition reduces naming latencies and affects brain responses, as indicated, for example, by decreased brain activity (repetition suppression; Desimone 1996; Grill-Spector et al. 2006; Schachter et al. 2007). It should be emphasized that while previous data make it likely that intention to speak and repetition determined faster activation, they do not indicate that intention to speak and repetition could have affected the temporal relationship of the various processes of naming. On the contrary, some MEG data (Matsumoto and Iidaka 2008; Friese et al. 2012) suggest that repetition affects multiple processes involved in naming, and thus, it is likely to leave the temporal relationship across these processes essentially unchanged.

Taken together these results lead us to tentatively propose phonological activation at about 150 ms. Accordingly, phonological and semantic activation would occur within similar latency ranges in picture naming. This result pattern is consistent with the Simultaneous Ignition hypothesis that proposes near-simultaneous and parallel activation of semantic and phonological features in picture naming. In contrast, the present results seem difficult to reconcile with the Semantic Priority hypothesis, under which semantic processing starts ahead of phonological processing.

### Why are Semantic and Phonological Information Simultaneously Accessed in Picture Naming?

One reason for proposing near-simultaneous phonological and semantic access at a latency of about 150 ms might lie in the nature of neuronal circuits in the brain. As shown by neurocomputational simulations, strong within-circuit connections may support an almost instantaneous ignition of neural activation, even within brain circuits involving distant cortical areas (for review, Pulvermuller et al. 2009). This sort of account could be extended to naming assuming a discrete functional circuit that connects, by strong neuronal links, neuronal subpopulations specialized for phonological and semantic processing, respectively. The phonological sub-circuits recruit perisylvian cortex, in particular superior temporal and premotor cortex, whereas the semantic sub-circuits engage overlapping and immediately adjacent fields in middle temporal gyrus and IFG. The close vicinity between these areas may account for the overlap in time we found with semantic and phonological variables. Even though future research will reveal a minimal temporal delay between the first neurophysiological indexes of access to semantic and phonological information in picture naming, such delay will likely be in the range of few milliseconds, not the range of 50 to 100 ms, as serial models of speech production have proposed.

A critical question concerns the plausibility of linking visual representations of objects to phonological representations of object names. Visual–semantic associations and visual–phonological associations are both vastly arbitrary and thus may raise equivalent challenges from a learning point of view. Nevertheless, having activation converging on word forms from 2 sources (visual and semantic) may facilitate and speed up word form selection—not a trivial advantage when we take into account the speed and accuracy demonstrated by adult speakers. But visual–phonological associations may be of considerably importance during language acquisition. While learning to speak, a child hears the names of visual objects or is explicitly taught those names as attention is drawn to specific objects in the visual scene. Associations between names and objects are often established even if the child's semantic knowledge is very rudimentary, if not absent. While visual-semantic associations often represent the only viable means supporting children's word learning, associations of this type might routinely form even in adulthood, where they serve to corroborate word learning and facilitate word retrieval.

Visually presented letters activate phonology in reading, a notion on which there is universal consensus across theories on reading (as an example, see Coltheart et al. 2001). The Simultaneous Ignition hypothesis proposes to extend this notion to object naming. Although the core assumption of the Simultaneous Ignition hypothesis is not novel, it is proposed here very tentatively, and it is therefore understood that further empirical support is required. Nevertheless, if confirmed, it will have several implications for neurocognitive models of naming. A first implication concerns the Semantic Priority hypothesis and the widely held claim that only semantics mediates access to word forms in picture naming. This claim should be revisited. Furthermore, the Simultaneous Ignition hypothesis would spur neuroanatomical research on neural structures underlying vision–phonology interface. Finally, from a developmental perspective, the Simultaneous Ignition hypothesis raises the possibility of a gradual change through language acquisition, with naming depending considerably on visual–phonological connections early in acquisition when semantics is rudimentary, but increasingly less as semantic knowledge grows and can thus provide a robust support to word retrieval.

### Early Brain Correlates of Word Form Processing

Previous research has identified a large network of brain areas supporting phonological processes, extending across temporal and inferior frontal cortex. Some proposals and results have suggested a role of the posterior superior temporal cortex and temporal-parietal junction (Hickok and Poeppel 2000, 2004), whereas others have highlighted the importance of anterior-temporal (Scott and Wise 2004) or temporal-lateral regions (Uppenkamp et al. 2006). Further proposals suggested that the middle or middle/superior temporal cortex processes lexical representations of word sounds (Damasio et al. 2004; Indefrey and Levelt 2004). On the other hand, the inferior frontal cortex, especially the posterior premotor part of Broca's area, has been related to phonological processes based on neuroimaging results (Vigneau et al. 2006) and recording of intracranial electrophysiology (Sahin et al. 2009), and inferior premotor cortex has been linked to articulatory information in both speech production and perception (Pulvermuller and Fadiga 2010). This wide range of regions could not be confirmed by the effects of the Word Form predictor, whose MEG correlates were observed only in left posterior middle temporal regions. Some of these localization results may be disputed because obtained with fMRI, which integrates activation over several seconds after picture presentation, but there are important exceptions. Intracranial recordings obtained while participants silently produced inflectional variants of visually presented words (as in walk → “walked”; Sahin et al. 2009) revealed modulation of electrophysiological responses related to word frequency in Broca's area at about 200 ms from word onset. Although comparisons between these data and those we obtained here should be drawn cautiously given the different eliciting stimuli (pictures vs. written words) and tasks (inflecting vs. naming), the data from Sahin et al. (2009) suggest activation of Broca's area reflecting word frequency at about 200 ms in word production, a result not revealed by the phonological/lexical regressor variable in our MEG study. It might be possible that the activation pattern we observed reflects the composition of the original variables composing the Word Form predictor (word length, number of phonological neighbors, and word frequency). Brain areas supporting word from encoding may be differentially sensitive to each of these phonological features, so that activation patterns may vary as a function of the tested features. This will remain an issue for future research. Furthermore, the use of overt naming task restricted our analyses within a 0- to 300-ms window; however, it is possible that effects of the Word Form variable appear at later latencies, as suggested by modulation of electrophysiological responses induced by word length that Sahin et al. (2009) observed at about 450 ms in Broca's areas and left superior temporal regions.

Finally, 2 caveats need to be addressed here. One caveat is that our ROIs did not cover the entirety of temporal and IFG areas that prior research has implicated in phonological processing (most notably, the anterior temporal cortex and the whole Broca's area). The other caveat is that the lack of perisylvian (including superior temporal) activation should not be interpreted too strongly, as superior temporal and middle posterior temporal ROIs are quite close together, touching on the spatial resolution limits of current MEG source localization.

### Early Effects of Semantics

A recent meta-analysis of functional neuroimaging studies requiring semantic knowledge (Binder et al. 2009) not only revealed a wide brain network implicated in semantic processing, but also sub-regions in the network sensitive to certain types of semantic information, including action knowledge (in supramarginal gyrus) and supra-modal semantic features (in middle and inferior temporal gyrus). Crucially, systematic investigation of semantic cortical activation firmly linked premotor and motor cortex activation to the access to knowledge about word-related actions (Hauk and Pulvermuller 2004; Boronat et al. 2005; Beuchamp and Martin 2007; Pulvermuller and Fadiga 2010; Rueschemeyer et al. 2010). Results from our ROI analyses align with these results in showing responsiveness to distinguishing semantic features and action knowledge in separate cortical regions (vPMC and IFG vs. inferior temporal gyrus). A novel contribution of our study is the demonstration that activation of these separate cortical regions occurs within the first 200 ms after the picture presentation that triggers naming processes. This finding is of particular relevance, as it indicates that action knowledge linked to objects and their related symbols is part of the information quickly activated upon availability of visual information. In addition, our results further confirm the activation of neural structures encoding functional action knowledge even in tasks, like picture naming, where action information is not explicitly required (Grafton et al. 1997; Hauk and Pulvermuller 2004; Saccuman et al. 2006; Rueschemeyer et al. 2010).

Our novel finding that activation related to action knowledge appears in frontal cortex before 200 ms has further implications for characterizing the brain network underlying picture naming. The pattern that has emerged from prior MEG studies of picture naming (Salmelin et al. 1994; Levelt et al. 1998; Vihla et al. 2006; Breier and Papanicolaou 2008; Hulten et al. 2009; Liljestrom et al. 2009; Pang and MacDonald 2012) has been one in which posterior cortex (temporal and inferior parietal) carries out the initial processing, whereas anterior cortex (especially the left prefrontal area) contributes at a much later point in time (typically ∼300 ms and up) computing the articulatory plans. Some of our data suggest a partially different time course: frontal areas processing key aspects of semantic knowledge, such vPMC and IFG that encode information on actions afforded by an object, become active almost simultaneously with other temporal areas implicated in semantic processing.

### Conclusions

A multiple linear regression approach was applied here for the first time in MEG. Results demonstrated good accord with temporal and spatial features observed with other neuroimaging techniques (ERPs and fMRI), a convergence that not only proves the reliability of the approach but also the suitability of this tool in neurocognitive research. We set out our MEG investigation of picture naming asking whether the brain correlates of word meaning and form processing, which we expected in left temporofrontal cortex, are separated in time and demonstrate earlier activation of semantics. In contrast to these expectations, we found indications of near-simultaneous activation of semantic and phonological processes. The Simultaneous Ignition hypothesis we discussed represents a tentative attempt to explain our data. The account assumes that visual processing instantaneously and simultaneously activates lexical circuits binding together the semantic and phonological knowledge about a given word. The first 200 ms appears to be crucial in word production, with critical processing of word meaning and word forms happening within this window of time. Our new results may be a key to a new understanding of how speakers produce words so effortlessly and efficiently.

## Funding

This work was supported by NIH (DC006242, MM), the Medical Research Council (UK) (MC-US-A060-0034 to FP; MC-US-A060-53144 to OH), and a Marie Curie Relocation Grant (MM). Funding to pay the Open Access publication charges for this article was provided by the UK Medical Research Council.
